# The Impact of Sports Training on the Spinal Cord Injury Individual’s Balance

**DOI:** 10.3390/s24237808

**Published:** 2024-12-06

**Authors:** Cristina Chieffo, Giorgia Chini, Tiwana Varrecchia, Irene Gennarelli, Alessio Silvetti, Vincenzo Molinaro, Ida Poni, Andrea Mariotti, Simone Tiberti, Annamaria Tamburro, Ilaria Calabrese, Sara Felici, Marco Bartoli, Loredana Gigli, Roberto Minella, Barbara Lucia, Aldo Toscano, Alberto Ranavolo

**Affiliations:** 1 Department of Occupational and Environmental Medicine, Epidemiology and Hygiene, Italian National Institute for Insurance Against Accidents at Work (INAIL), Via Fontana Candida 1, 00078 Monte Porzio Catone, Italy; c.chieffo@inail.it (C.C.); g.chini@inail.it (G.C.); al.silvetti@inail.it (A.S.); v.molinaro@inail.it (V.M.); a.ranavolo@inail.it (A.R.); 2 Human-Robot Interfaces and Physical Interaction Laboratory, Italian Institute of Technology, 16163 Genoa, Italy; irene.gennarelli@iit.it; 3 Centro Protesi INAIL Rome Branch, Ospedale C.T.O. Andrea Alesini, Via San Nemesio, 21, 00145 Rome, Italy; i.poni@inail.it (I.P.); a.mariotti@inail.it (A.M.); a.toscano@inail.it (A.T.); 4 ASL Roma 2, Ospedale C.T.O. Andrea Alesini, Via San Nemesio, 21, 00145 Rome, Italy; simone.tiberti@aslroma2.it (S.T.); annamaria.tamburro@aslroma2.it (A.T.); ilaria.calabrese@aslroma2.it (I.C.); sara.felici@aslroma2.it (S.F.); marco.bartoli@aslroma2.it (M.B.); 5 ASL Roma 3, Ospedale C.P.O. Centro Paraplegici Gennaro di Rosa, Viale Vega 3, 00122 Rome, Italy; loredana.gigli@aslroma3.it (L.G.); roberto.minella@aslroma3.it (R.M.); barbara.lucia@aslroma3.it (B.L.)

**Keywords:** balance, optoelectronic system, kinematic analysis, spinal cord injury, sport rehabilitation, sway density curve, sway length, wheelchair sports

## Abstract

Spinal cord injury (SCI) causes major challenges to mobility and daily life activities and maintaining balance becomes a crucial issue. Individuals with SCI often need to adopt new strategies to manage balance with minimal discomfort. Sports and physical activities have become one of the most popular rehabilitation methods for people with SCI. The assessment of balance improvement currently relies on subjective evaluation scales, and this study aims to quantitively assess the efficacy of sports on the balance strategies of people with SCI. Twenty-two SCI people remained seated still for 30 s, with their eyes open and closed, and we recorded trunk kinematics with an optoelectronic system before and after a three-months sports program. We also computed trunk total sway length, mean velocity, and sway density curve. Statistical analyses were performed to compare SCI people before and after the rehabilitation program and to investigate any correlations between the trunk balance parameters and the clinical scales. The results demonstrate improvements in static balance, with significant reductions in sway length and mean velocity. In conclusion, our findings confirm the potential of sports to enhance balance in SCI individuals and suggest that integrating structured sports programs into rehabilitation can improve stability and postural control.

## 1. Introduction

Spinal cord injury (SCI) presents significant challenges to individuals, impacting their mobility and daily life activities [1]. The functionality of muscles of the upper or lower limbs may be impaired according to the level of SCI and the completeness or the incompleteness of the lesion [2].

One of the challenges that people with SCI may face is maintaining both static and dynamic sitting balance. The former characterizes the performance of the control system in a static position during quiet sitting while the latter characterizes the response of the postural control system to an applied perturbation [3]. Very often, people with SCI are unable to establish the balance they had before the injury, so they reorganize their body districts’ behaviors to achieve adaptive postures that cause them less pain [4].

Among all the rehabilitation strategies, sports and physical activity play a fundamental role for people with SCI, not only for their physical well-being, as the cardiovascular and muscular benefits demonstrate [5], but also for improving balance and stability [6,7]. Sports and physical activities provide a practical context in which some adaptive strategies can be developed, practiced, and refined. Sports differ from and are one more tool than therapeutic exercise. In fact, although therapeutic exercise and sports have common characteristics, sports allow for work on general and special coordination skills. Specifically, in terms of general coordinative skills, motor control ability is increased because sports allow for movements to be performed according to the respective goals set for each sport. The precision needed to achieve the goals is the most important factor. In terms of special coordinative ability, on the other hand, we have fine dexterity, proprioceptive differentiation, and oculo-motor coordination.

Numerous studies have demonstrated that physical activity and sports are powerful tools for preventing a crippling cycle that people with myelopathy frequently experience. This cycle causes them to live in a state of forced inactivity that exacerbates and prolongs problems such as oxidative stress, osteoporosis, chronic systemic inflammation, increased fat mass, sarcopenia, decreased cardiovascular efficiency, insulin resistance with increased atherosclerotic cardiovascular risk, dyslipidemia, and ultimately, early death [8]. After undergoing a clinical and functional evaluation by qualified health professionals, SCI individuals who engage in moderate-intensity or higher physical activity for at least 30 min each session, three days a week, improve cardiovascular function, lower the risk of osteoporosis and metabolic syndrome, counteract the detrimental effects of a sedentary lifestyle on cardiovascular risk, increase muscle strength, break the vicious cycle of sedentary–hypotrophy–pathology–menopause, and prevent disease onset even more effectively than the general population [9,10,11,12,13]. Additionally, the scientific literature indicates that a person with tetraplegia who regularly exercises enhances their muscle strength, endurance, fitness, and fatigue tolerance, all of which have a major influence on their ability to move around in a wheelchair [14]. Moreover, increased mobility fitness improves everyday functioning, including transitioning between a wheelchair and automobile, bed, or bathtub [14,15]. A further advantage of physical exercise over other types of therapy is that it can enhance fatigue tolerance, endurance, strength, coordination, and fitness in SCI people [16]. In addition to improving wellbeing, engaging in different types of physical exercise increases muscular strength and speeds up adaptation to disability [17,18,19,20].

By engaging non-postural muscles for balance control, individuals learn to compensate for the loss of lower limb function, enhancing their stability and mobility in daily life [21].

There are several studies in the literature confirming that the involvement in sports of people with SCI has a positive association with their rehabilitation, increases their independence, gives them greater self-confidence, improves their psychological well-being and quality of life, and gives them aspiration for further development and community integration [22,23,24].

To understand the real effectiveness of a sport rehabilitation program on improving the balance of people with SCI, a collaboration between the Italian Paralympic Committee, Italian National Institute for Insurance against Accidents at Work (INAIL), and the Local Health Authority (ASL Roma 2 and ASL Roma 3) was born through the “Piano Individuale Terapeutico Sportivo (PITS)” project. This consists of bringing people with SCI closer to the sports world by allowing them to practice free of charge, for three months, two times a week, six different sports: archery, tennis, swimming, athletics, fencing, and bowls, with the hope they will choose a sport to practice permanently.

Clinicians routinely use both scales and objective measurement methods to assess a subject’s balance, particularly after rehabilitation programs. Commonly employed scales include the Berg Balance Scale, the Walking Index for Spinal Cord Injury (WISCI II), the Spinal Cord Independence Measure (SCIM), the 10 m walk test (10 MWT), and the Timed Up and Go test (TUG) [25]. While these scales are widely used, they are often subjective and rely on the clinician’s interpretation.

To complement these subjective methods, clinicians increasingly incorporate objective measurement techniques such as human motion capture systems. These include optoelectronic systems, surface electromyography, force platforms, and wearable sensors, which provide non-invasive, objective data on human body kinematics and kinetics [26,27]. Among these, stabilometric measurements are especially common. They involve estimating parameters related to the displacement of a subject’s center of pressure (COP) using instrumental tools [28]. The COP is the vertical projection of the body’s center of mass (COM), and balance is maintained when the COP remains within the base of support provided by the subject’s feet. The study by Romano et al. [29] also demonstrated a high correlation between stabilometric parameters derived from the COP and those calculated from the center of mass, indicating that both approaches provide equally robust stabilometric information. Therefore, the posturography assessment of people who cannot stand can also be evaluated by means of COM displacement analysis.

This observational study aims to understand how much and in what way the balance strategies of people with SCI are changed after a sport rehabilitation program. To reach this aim, data were recorded from the INAIL prosthesis center branch in Rome during the PITS project. The subjects performed sports activities externally in the facilities provided by the Italian Paralympic Committee and then routine stabilometric parameters and clinical scales were acquired. We collected personal information, clinical records, and balance performance for all subjects and studied the differences pre–post sport treatment. We hypothesize that a sport rehabilitation program improves the ability of people with SCI to maintain static balance.

## 2. Materials and Methods

### 2.1. Subjects

This observational study made it possible to analyze data from patients followed by the centers involved in the project over a year. The clinical and instrumental evaluations were carried out independently of the project because they were part of the services provided. Subjects performed some sporting activities externally as described below.

Twenty-six people with SCI participated in the activities (21 Male and 5 Female; Age: 44.58 (±13.67) years; Height: 1.75 (±13.17) m; Weight: 74.35 (±13.17) kg; BMI: 24.33 (±4.33) kg/m^2^; Time since the injury: 11.88 (±10.94) years). One subject did not participate in the T1 acquisitions, and instrumentation problems led to the exclusion of three other subjects. So, data could be acquired and analyzed for twenty-two subjects (17 male and 5 female; age: 45.26 (±14.33) years; height: 1.75 (±0.09) m; weight: 75.39 (±12.28) kg; BMI: 24.60 (±4.04) kg/m^2^; time since the injury: 12.68 (±11.13) years). No complications arose during the period of sport rehabilitation program, except for one subject who experienced functional overuse of the left shoulder and returned to the practice after two weeks of rest.

All the analyzed characteristics of the people with SCI are schematized in Table 1. We included both acute subjects within one year of injury and late subjects on clinic, then outpatients. Since most of those enrolled were chronic, their weight was also in the normal range, having already gone through the first acute phase that usually leads to underweight.

In addition to the biological people characteristics, the levels of the injury on the spine were also listed; the AIS or American Spinal Injury Association (ASIA) Impairment Scale [30,31]; and the years since the injury.

The AIS is a standardized tool used to assess and classify the severity of spinal cord injuries. There are four grades: A––Complete Injury, no sensory or motor function is preserved; B––Sensory Incomplete, sensory present but no motor function is preserved; C––Motor Incomplete, motor function is preserved and more than half of key muscles below the neurological level have a muscle grade less than 3; D––Motor Incomplete, motor function is preserved below the neurological level, and at least half of the key muscles below the neurological level have a muscle grade greater than or equal to 3; E––Normal [32].

### 2.2. Clinical Scale

The scales collected during clinical routine are listed and described below:

1. The Spinal Cord Independence Measure (SCIM) ranges from 0 to 100, representing a person’s ability to perform daily living activities. A higher score indicates greater independence, with a focus on personal care movements and respiration control [33]. It measures three domains: Self-Care, Respiration and Sphincter Management, and Mobility. The Mobility domain specifically evaluates trunk activity, which is divided into static trunk, dynamic trunk, trunk activity, and wheelchair mobility. Specifically:
  - TRUNK Static: the capacity to maintain a stable and balanced trunk position while sitting without additional support or movement.
  - TRUNK Dynamic: the ability to control the trunk during movements, such as reaching, bending, and twisting, which are crucial for performing various functional tasks.
  - TRUNK Activity: combines both static and dynamic control, emphasizing the overall functionality and independence of the trunk in various daily activities.
  - WHEEL: assesses the individual’s ability to move and control a wheelchair independently, including indoor/outdoor mobility, ramp use, and inclined surfaces, which is crucial for those with limited lower limb function.
2. The Disabilities of the Arm, Shoulder, and Hand (*DASH*) scale is a standardized assessment tool used to assess the functional status of individuals with upper extremity musculoskeletal disorders, including the shoulder, arm, and hand, and to measure treatment outcomes [34]. It is a self-assessment questionnaire with multiple items rated from 1 to 5. The *DASH* score is calculated as follows:
  (1) $$DASH=\left(\frac{\sum Response\;score-1}{n-1}\right)\times 25$$
  where *n* is the number of completed items (at least 27 out of 30 must be answered). A score of 0 indicates no disability, while a score of 100 indicates the maximum possible disability.
3. Moorong Self-Efficacy Scale (MSES) is a psychological assessment instrument designed to measure self-efficacy, i.e., confidence in one’s ability to handle specific situations, in people with physical disabilities, particularly those with spinal cord injuries. Likert scale ranging from 1 (very uncertain) to 7 (very certain). The highest scores on the MSES suggest high autoefficacy or stronger beliefs in people able to control their behavior and results, such as personal hygiene, home participation, maintaining relationships, and access to community and recreational activities [35].

### 2.3. Kinematic Recordings

The optoelectronic system used is the BTS Bioengineering SMART DX 6000 system (BTS, Milan, Italy) with eight infrared cameras (sample frequency 340 Hz) capable of tracking the trajectory of retro-reflective markers in a calibrated area.

Each participant was equipped with three spherical markers (15 mm in diameter) covered with aluminum powder reflective material placed over the cutaneous projections of the spinous processes of the seventh cervical vertebrae and, bilaterally, over the acromion.

### 2.4. Test Procedures

The assessments took place in the motion analysis laboratory of the INAIL Prosthetic Center in Rome branch in the Andrea Alesini Orthopedic Trauma Centre Hospital in Rome-ASL Roma 2.

The test consisted of sitting still for 30 s or as long as the subjects could [36,37]. The subjects repeated the test twice, with open ryes (OE) and with closed eyes (CE) to assess the contribution of the visual system to maintaining equilibrium [15].

Subjects were asked not to lean on the back of their wheelchair and to have a comfortable self-chosen posture to not affect the balance measurement.

We measured our primary outcome on a “before the sport rehabilitation program” observation in March 2023 (T0) and an “after the sport rehabilitation program” observation in April 2024 (T1) during the people’s clinical routine.

### 2.5. Sports

All the subjects practiced, free of charge and two times a week for three months, the following sports: archery, tennis, swimming, athletics, fencing, and bowls.

The six sport activities were performed with the constant support and supervision of trained instructors. In general, athletics and bowls were intended to develop functional muscular resistance to wheelchair pushing. Tennis, fencing, and archery were included in the training program to improve trunk balance, hand–eye coordination, and strengthening of the upper limbs. Swimming was intended to provide a global strengthening of all the respiratory and residual muscles of the body [38,39].

Sports sessions rotated monthly, allowing participants to enjoy a variety of activities. Each subject each month played two sports twice a week for the duration of the two-hour session. Specifically, one month they practiced swimming and tennis, another month bowls and fencing, and another month athletics and archery. More in detail, for all the practiced sports, the exercises performed during the sports rehabilitation program were integrated with warm-up routines before sports sessions to reduce the risk of injury. Following evidence-based guidelines, during these warm-up sessions participants performed moderate-intensity aerobic exercise and strength training. Each warm-up session included 20–30 min of aerobic activity combined with strength exercises, performed in three sets of 8–10 repetitions at 50–80% of their maximum strength for major muscle groups [9,40,41]. These exercises helped improve fitness, strength, and decrease spasticity by increasing articulacy.

After the warm-up, SCI individuals engaged in all sports with the same regulations as their non-disabled counterparts, utilizing specially designed workouts [42]. Training loads were based on their musculoskeletal, respiratory, and cardiovascular fitness. People with SCI naturally incorporate kinesitherapy (corrective, individualized, and unique exercises relevant to each athlete’s functional state) into their sports training activities in addition to standard training methods [42].

More in detail, as regards swimming, the training adhered to the Halliwick Concept [43], which is one method of exercising in water created by the British Association of Swimming Therapy. This approach to teaching swimming and water therapy focuses on teaching students how to move and remain in the water freely and safely. This is accomplished by managing breathing, head motions, and balance. SCI people can learn how to stay balanced in an unstable environment by doing these activities. When stability (balance) is attained, regulated movement may start, which helps the SCI people become more independent, has a multilateral effect on the body, decompresses the spine, and strengthens the muscles, circulatory, and respiratory systems [43]. Regarding fencing adapted to the needs and abilities of people with SCI, the exercises consisted in strengthening the muscles in the upper limbs, back, and abdomen, technical and tactical drills, and participation in challenges and duels. Tennis incorporated both racket and wheelchair movements that are symmetrical for both limbs and can be energetic, resistive, or isometric. It is a balancing workout because the motions used to smash a ball with a tennis racquet throw off the body’s equilibrium (thus increasing body control and comprehension of body alignment in space) [44]. Athletics included postural gymnastics exercises that targeted upper body and core strength, such as bicep curls, shoulder press, bench press, Russian twists, distance, and wheelchair races. As regards to archery, it involved workouts that encourage the activation of the muscles in the upper limbs, as well as the back, abdominal, trunk, and head. Throwing the arrow at targets at various locations and distances, technical and tactical exercises, and playing training and competitive games were all part of the training. Lastly, bowls included exercises focused on strengthening the upper body, increase dynamic stability, perceptual–motor awareness, and visual–motor coordination. Moreover, the bowls training also included technical and tactical exercises, throwing balls to various targets, and playing games for practice and competition [45].

### 2.6. Data Analysis

The raw data acquired during the experiment by the SMART capture software (Version 1.10.470.0, BTS Bioengineering, Milan, Italy) were exported to a *.tdf* file and subsequently imported into SMART tracker software (Version 1.10.470.0, BTS Bioengineering, Milan, Italy). Each marker was labelled according to a customized protocol to track it in each time frame. At this point, the *.tdf* file was read by the SMART Analyzer software (Version 1.10.470.0, BTS Bioengineering, Milan, Italy), which allows for an initial processing of the data. Smooth filtering, linear interpolation, and cutting into intervals of 30 s were applied to the raw data. Then, the data were processed to obtain the parameters useful for this study using an ad hoc MATLAB (Version R2022a, 9.12.0.2170939, MathWorks, Natick, MA, USA) code.

#### Center of Upper Body and Balance Indices

**Figure 1:**
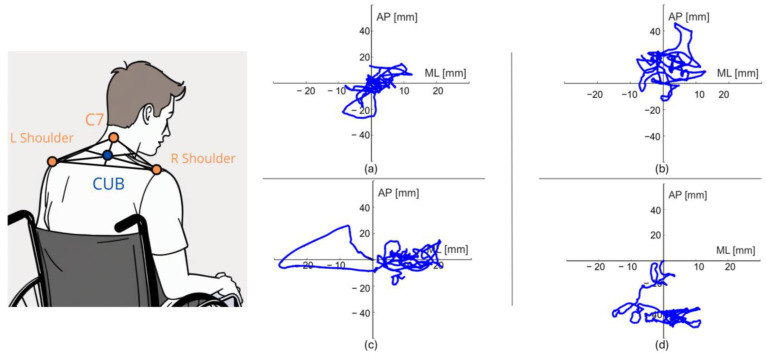
On the left: a drawing showing how the center of upper body (CUB) is constructed. On the right: example of sway path in the four conditions considered. (**a**) Time T0 eyes open; (**b**) Time T1 eyes open; (**c**) Time T0 eyes closed; (**d**) Time T1 eyes closed.

We defined and monitored the center of upper body (CUB) to assess the balance in a wheelchair. Geometrically, this point was constructed as the barycenter of the triangle formed by the three used markers. The displacement of the postural ball on the plane of the ground (sway path) and their anteroposterior (AP) and mediolateral (ML) oscillations were used as an index of the maintenance of balance in people with SCI. An illustrative figure of a subject’s sway path is shown in Figure 1.

**Figure 2:**
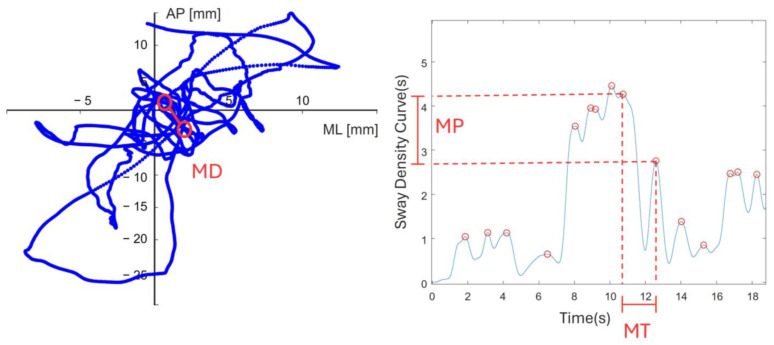
A graphical definition of the three parameters extracted from the sway density curve. On the left, the mean spatial distance of peaks (MD) on the sway path; on the right, the mean time interval (MT) and the mean value of the peak (MP).

For both conditions, OE and CE, the following parameters concerning the CUB were evaluated [46]:

- Total Sway Length (*TSL*) of the CUB’s path during time (mm):
  (2) $$TSL=\overset{N-1}{\underset{n=1}{\sum }}\sqrt{{\left(AP\left[n+1\right]-AP\left[n\right]\right)}^{2}+{\left(ML\left[n+1\right]-ML\left[n\right]\right)}^{2}}$$
  where N represents the total samples equal to task duration multiplied by sampling frequency (30 × 340 = 10,200 samples).
- Mean Velocity (*MV*) as the average velocity of the sway path (mm/s):
  (3) $$MV=\frac{TSL}{T}$$
  where T is the total duration of the test in seconds (30 s).
- Sway Density Curve (SDC): illustrates the elapsed time series, indicating the CUB inside a circle with a 2.5 mm radius [47]. The time series of SDC were filtered by a low-pass Butterworth filter, fourth-order and cutoff frequency 2.5 Hz [48]. Peaks on this curve signify temporary postural stability; conversely, the valleys on the curve indicate transitions between these moments of stability [49,50]. Three parameters were extracted from each SDC (Figure 2):
  ○ MT = mean time interval between successive peaks (s);
  ○ MP = mean value of the peaks (s);
  ○ MD = mean distance between successive peaks (mm).

### 2.7. Statistical Analysis

The statistical analysis was performed using MATLAB (Version R2022a, 9.10.0.2015706, MathWorks, Natick, MA, USA). Preliminarily, the Shapiro–Wilk normality test was applied to check for normal distribution. Depending on the results of the normality test, the paired *t*-test or the Wilcoxon test was used to identify any major differences in the above-described balance parameters before and after the sport rehabilitation program and between the OE and CE condition, both at T0 and T1.

Pearson’s test with PASW Statistics software (Version 17.0.2 2009) was used to investigate any correlations between various demographic, clinical, and functional parameters related to SCI before and after the sport rehabilitation program.

The last two analyses were made to investigate potential differences in balance change before and after the sport rehabilitation program by dividing the sample of people in clusters; one according to sense–motor capacities preserved after injury and a second one based on the spinal cord level of the lesion.

For the first one, the sample was divided into two clusters, one to which the people with AIS grade A and B injuries belonged (14 people) and the other with grades C and D (7 people).

For the second one, the two clusters were made of 8 people for the level of injury that includes the fourth dorsal vertebrae up to the cervical vertebrae and 13 people including all the sacral and lumbar vertebrae up to the fifth dorsal vertebra.

The statistical analysis in each case was carried out by taking into consideration the deltas defining the changing of a certain balance parameter. The definition is schematized in the equation below as an example of a generic parameter:

(4) $$\Delta =\left|Parameter_{T1}-Parameter_{T0}\right|\;$$

The independent samples *t*-test was applied if the data were normally distributed and the two-sided Wilcoxon rank sum test if they were not normally distributed. Following this same logic, considering the deltas of the parameters, a comparison was made between OE and CE conditions within the clusters. In those cases, the statistical difference was performed using *t*-tests for paired samples when normal distributions or Wilcoxon signed rank tests when non-normal.

The significance level for all statistical analyses was set at *p*-value < 0.05.

## 3. Results

### 3.1. Comparison T0–T1 and OpenEyes-ClosedEyes

**Figure 3:**
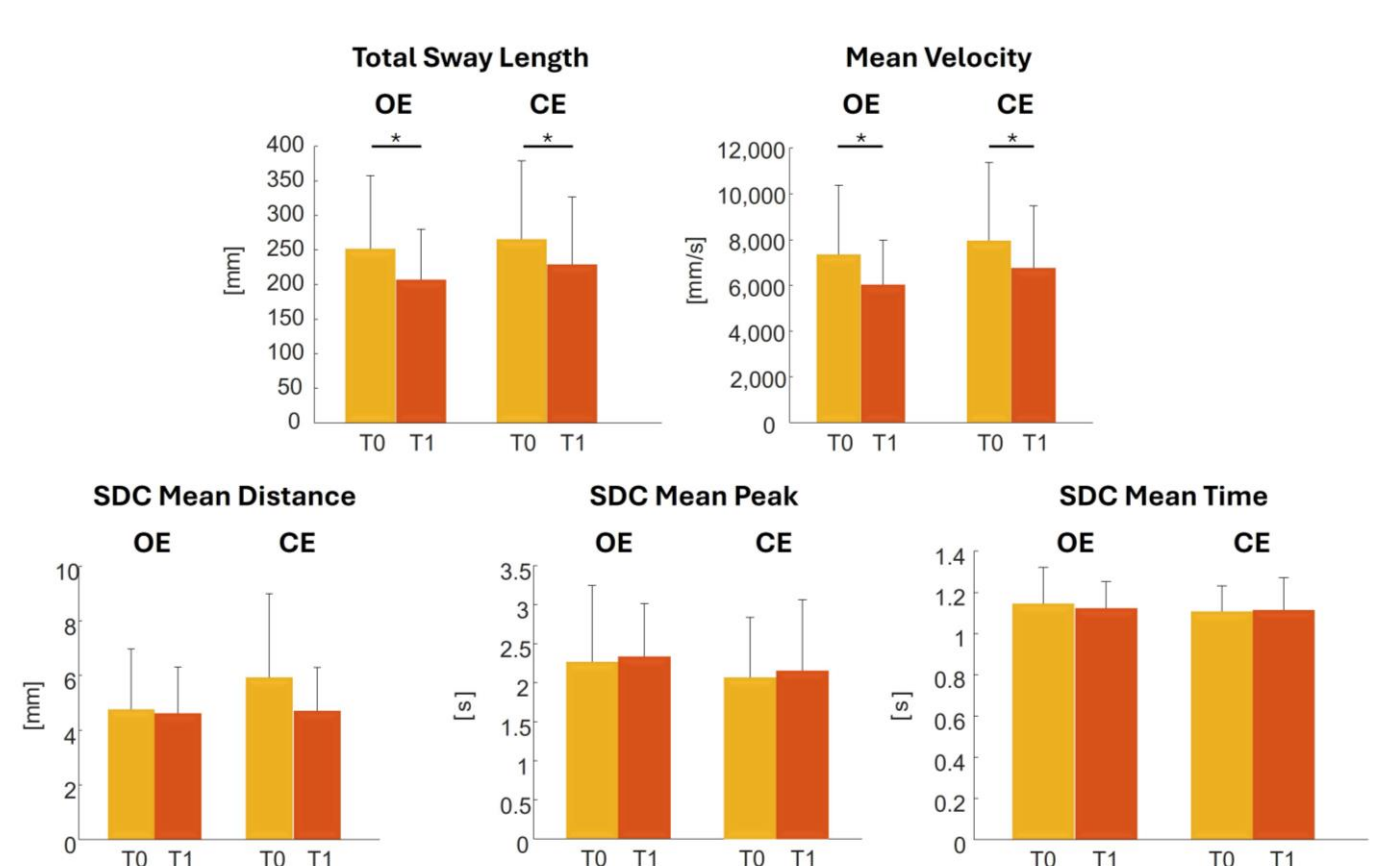
Mean and standard deviation values of the total sway length, mean velocity and mean distance, mean peak and mean time of the sway density curve’s peaks indices in the conditions analyzed before (T0) and after (T1) sport rehabilitation program with both eyes open (OE) and closed (CE). Data are represented as bar plots with error bars indicating variability. Metrics are given in units of millimeters [mm] and seconds [s]. * Level of significance 95% when *p* < 0.05.

The mean and standard deviation values of the investigated parameters are reported in Figure 3. Statistical differences were also reported in Figure 3: the total sway length (*OE: *p* = 0.04; CE: *p* = 0.03) and mean velocity (*OE: *p* = 0.04; CE: *p* = 0.02) significantly decrease from T0 to T1.

### 3.2. Correlation Index-Clinical Scales

**Figure 4:**
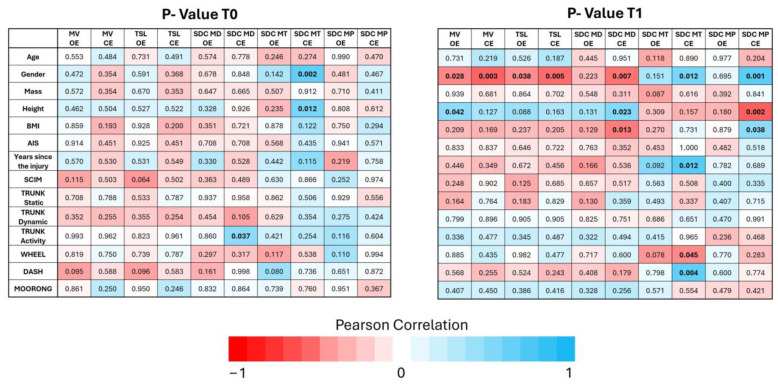
Pearson correlation coefficients and their corresponding *p*-values at two different time points (T0 at left and T1 at right) for the index in relation to demographic data and clinical measures in both conditions (OE and CE). Bold *p*-values less than 0.05 indicate statistically significant correlations. Blue shades indicate positive correlations, red shades indicate negative correlations, and the intensity of the color represents the correlation’s strength.

The correlations between demographic, clinical, and functional indices are shown in Figure 4.

At time T0, significant positive correlations were found between gender (*p* = 0.002) and height (*p* = 0.012) with SDC MT CE condition and between trunk activity and SDC MD CE condition (*p* = 0.037).

After the sport rehabilitation program, gender demonstrated negative significant correlation with the MV OE (*p* = 0.028), MV CE (*p* = 0.003), TSL OE (*p* = 0.038), TSL CE (*p* = 0.005), SDC MD CE (*p* = 0.007), and a positive one with SDC MT CE (*p* = 0.012) and SDC MP CE (*p* = 0.001) conditions.

Also, height characteristic presents a positive significant correlation with MV OE (*p* = 0.042), SDC MD CE (*p* = 0.023), and a negative one with SDC MP CE (*p* = 0.002). A significant *p*-value is stated also for the correlation of the BMI and SDC MD Eyes Closed (*p* = 0.013) and with SDC MP CE (*p* = 0.038).

Years since injury demonstrated positive significant correlation with the SDC MT CE condition (*p* = 0.012).

In contrast, a strong negative correlation was observed between WHEEL score and the SDC MT CE condition (*p* = 0.045), while a strong positive correlation was found between the DASH score and the same balance index (*p* = 0.004).

### 3.3. Comparison Sense–Motor Capacities

The corresponding statistical analysis performed on the deltas between the two moments T0 and T1 is schematized in Table 2.

In TSL, group AB demonstrates a statistically significant reduced CE condition (*p*-value = 0.0339). The comparison ∆AB-∆CD in the CE condition also shows a significant difference (*p* = 0.0271).

In MV in both OE and CE, the difference between groups is significant (*OE = 0.0327; CE = 0.0398). A significant difference is present in the comparison of OE-CE for group CD (*p* = 0.0101).

The last significance is present for the changing of SDC mean distance in group CD in the OE condition (*p* = 0.03647).

### 3.4. Comparison Level of the Lesion

The corresponding statistical analysis performed on the deltas between the two moments T0 and T1 is schematized in Table 3.

In this case, the inter-group difference is statistically significant for the TSL in the closed eyes condition (*p* = 0.0370. For the MV instead, the main differences are present for the C-D4 group between the two visual conditions (*p* = 0.0156) and between the two groups without the use of visual input (*p* = 0.0367).

A last main result is for the SDC MT parameter who presents a *p*-value equal to 0.0105 for the CE condition between groups.

## 4. Discussion

The purpose of this observational study was to assess the static balance ability of people with SCI both before and after participating in a sports rehabilitation program. Both the clinical scales and objective evaluations of static balance kinematics, as part of the subjects’ routine clinical care, were performed using questionnaires and an optoelectronic system that tracks the movement of passive markers during the test, respectively.

Indices commonly used in posturology were calculated both before and after the sports rehabilitation program, as well as during tests performed with eyes open and eyes closed. The statistical differences between these indices were then estimated.

The general trend of all indices shows an improvement in stability and balance maintenance when following sport rehabilitation program, as already demonstrated in other studies on different sports cases [7,21]. Even though all standard deviations are high when dealing with pathological people, at time T1 a smaller interval around the mean can be seen. Particularly, the total sway length and mean velocity measures decrease from T0 to T1 under both open and closed eyes conditions. The presence of significant reductions in both eye conditions suggests that these improvements are robust and not solely dependent on visual input. Other measures like sway density curve mean distance, mean peak, and mean time do not show significant changes, indicating that the improvements are more pronounced in some aspects of stability than others, aligning with findings that these parameters may not capture the full complexity of postural stabilization processes [47].

A correlation was made between the indices chosen for the study and the clinical scales obtained from the subjective evaluation of the clinical specialists.

The variables exhibit significant correlations at T0 and T1, with some showing consistent patterns. Three factors seem to have a positive influence on the effectiveness of the sports rehabilitation program, namely fewer years elapsed since the injury, shorter height of the subject, and being of the female gender.

This suggests that gender differences might play a role in balance performance with individual’s eyes open and closed after the rehabilitation program. Studies show that females may exhibit superior balance learning compared to males, especially in tasks that require maintaining balance with both eyes open and closed. This suggests the possibility of more tailored rehabilitation approaches based on gender, which could enhance effectiveness, particularly when considering these balance dynamics [51,52].

Additionally, the trunk activity presented a significant relationship with the sway density curve mean distance eyes closed condition (*p* = 0.037), indicating that, even if trunk activity from the clinical scales is well present, when visual input is unavailable, maintaining balance is difficult prior to treatment.

The upper limb functionality, quantified using the DASH scale, is a critical determinant of performance in maintaining balance with eyes closed, providing insight into targeted therapeutic approaches.

These observations after following the sport program highlight how certain variables consistently influence muscle function and limb mobility over time, emphasizing the importance of considering multiple factors in assessing and managing spinal cord injuries. Over time, certain correlations become more pronounced or diminish, suggesting a dynamic interaction between these variables and muscle functionality in individuals.

Subsequently, an analysis was carried out dividing the subjects into two clusters: one that includes subjects with no sensor-motor capacities, AIS grade A, or with only sensory skills, AIS grade B, and the other group with sense–motor capacities still present but with different intensities, classified as grade C and D. The comparison was made between the deltas of the balance indices, defined as the difference between two time points, T0 and T1.

In terms of total sway length, group AB demonstrates a statistically significant difference in sway when visual input is removed. Therefore, the decrease in this parameter and consequently the post-rehabilitation improvement in balance is greater in the open-eye condition. This could be explained by the fact that achieving an improvement in balance even without visual input is very complex, as also demonstrated by Rodriguez et al. [53], in the case of athletes with chronic ankle instability. Vision plays a crucial role in maintaining balance, providing constant feedback on body orientation relative to the environment. In people with SCI, the visual capacity takes an even more important role considering the difficulties in trunk control. Without it, the body must rely solely on internal cues from the vestibular system (inner ear) and proprioception (muscle and joint sensors), which can be less reliable, especially in pathological conditions. The inter-group comparison in same CE conditions also shows a significant difference, so there was a greater change in the TSL parameter for the CD group, thus reporting an improvement in the equilibrium of people with spinal cord injury when few motor functions are still preserved compared to the AB group in which no motor functions below the lesion are present.

Mean velocity presents, in both visual conditions, a high difference between groups, especially in the CE condition. This indicates that group CD improves most with visual input after the sport rehabilitation program, which is reflected in the higher delta mean sway velocity. The significance in the comparison of OE and CE for group CD supports this observation.

For SDC mean distance, AB- CD demonstrates significant difference in the OE condition, while no significant change is seen in the CE condition. This could imply that the CD group compensates better when visual feedback is available but is less adept when visual cues are removed than for the AB group. The other parameters, such as mean time and mean peak, show no significant differences across groups and conditions, indicating these measures might be less sensitive for detecting balance changes in these circumstances.

The last analysis was conducted by dividing the subjects into two groups: one that includes individuals who have a spinal cord injury involving high vertebrae, thus the fourth dorsal vertebrae up to the cervical vertebrae, and the second whose lesion involves the lower vertebrae which means all the sacral and lumbar vertebrae up to the fifth dorsal vertebra.

In terms of total sway length, a statistically significant difference is the one between the two clusters of subjects in the closed-eye condition. It can therefore be seen that the decrease in the parameter and thus the improvement in balance is more pronounced in persons with a lower spinal cord injury (D5-L). The same trend is also confirmed by the mean velocity. The significant difference in this case is in the group with the higher lesion, which shows a clearer post-treatment improvement in the open-eye condition and in the comparison between the two groups in the closed-eye condition.

The last parameter to focus on is the SDC mean time, which shows for the closed-eye condition a significant difference between the two groups of subjects. The temporal distance between the stability peaks of the sway density curve decreases by about twice as much for the group with high spinal cord injury post-treatment.

Unfortunately, as stated in the introduction, there is very little scientific literature on the evaluation of the kinematic behavior of the trunk in people with SCI. To the best of the authors’ knowledge, only one study compared balance biomarkers derived from IMUs’ readouts under conditions that challenged balance by affecting somatosensory (i.e., standing on hard vs. foam surfaces) and visual (i.e., eyes open vs. closed) inputs [54].

From this study emerged that, due to impaired somatosensory feedback, individuals with incomplete SCI showed a higher and lower reliance on visual and somatosensory information with respect to healthy individuals, respectively, for maintaining balance.

This comparison to the literature, on the one hand, confirms our findings assessing the effect of visual feedback in maintaining balance in people with SCI. Moreover, the coherence founded with the literature on this topic reinforces our findings about the necessity to expose people with SCI to rehabilitation with specific trunk exercises.

This study demonstrated a common trend for all indices of improvement in the maintenance of balance in the time following the sport rehabilitation program. Although in some cases the statistical significance was not demonstrated due to possible interpersonal differences between pathological people. Supporting other previous research [22], it was shown that sport has a positive effect on postural balance and that activity, even if only for three months, leads to greater body stabilization.

One notable drawback of this study is the relatively small number of subjects recruited in the study sample, which limits the ability to generalize our findings. On the other hand, spinal cord injury is to be considered, with respect to some neurological motor diseases such as stroke or Parkinson’s Disease, a rare pathology in terms of incidence and prevalence. Indeed, even if the current sample size is limited, increasing the sample could strengthen the results obtained. Moreover, another limitation of this study is that it was conducted on a sample consisting mainly of males, so in the future we might consider extending the study by expanding it to include more of the female population to assess any gender differences.

Another limitation is attributable to our proposed methodology, which is based on the CUB study referring only to the upper body and the cervical region, not considering the distal parts of the spine. In future experimental studies, it would be interesting to evaluate the integration of wheelchairs with pressure sensors to gain information about force distribution in the wheelchair during balance maintenance, offering a more comprehensive understanding of biomechanical adaptations in SCI individuals.

Additionally, the study’s inclusion of many sports disciplines is another limitation because it may overrepresent some results and hinder the correlation between various treatments and spinal cord injuries. To further understand the unique contributions of each intervention technique in the rehabilitation of spinal cord injuries, more research that focuses on specific sports disciplines with a more carefully designed experimental approach would be beneficial. Finally, a further limitation of this study lies in the evaluation of the effect of sports therapy only in the short term. Therefore, in the future, surely it may be of interest to conduct studies that include longer-term sports rehabilitation programs, and which can also conduct interim evaluations to ascertain progress over time and the ability to maintain functional recovery.

## 5. Conclusions

In conclusion, this study demonstrates the significant potential of sports-based rehabilitation programs to enhance balance in individuals with spinal cord injuries. The findings showed general improvements in stability and balance maintenance, which support the value of sports as a rehabilitative tool, even within a relatively short timeframe.

## Figures and Tables

**Table 1:** Each subject’s physical and biographical characteristics.

Subject	Age (Years)	Gender	Mass (kg)	Height (m)	BMI (kg/m^2^)	Level of Injury	AIS Grade	Time Since the Injury (Years)
S001	43	M	93	1.90	25.76	D4	D	1
S002	50	M	63	1.75	20.57	D6	A	22
S003	50	M	62	1.69	21.71	C5	B	6
S004	34	F	57	1.68	20.20	D8	A	3
S005	40	M	82	1.85	23.96	C6	B	12
S006	59	M	83	1.70	28.72	D7	A	14
S007	70	M	78	1.66	28.31	D10	D	2
S008	48	M	90	1.90	24.93	D7	D	29
S009	54	M	71	1.80	21.91	D11	A	18
S010	54	M	80	1.78	25.25	D10	A	18
S011	20	M	78	1.72	26.36	D1	D	2
S012	32	M	60	1.80	18.52	L2	B	4
S013	35	F	76	1.58	30.44	-	-	35
S014	25	F	53	1.60	20.70	D3	A	4
S015	42	M	90	1.85	26.30	D3	A	12
S016	42	M	65	1.90	18.01	D8	D	15
S017	71	M	89	1.75	29.06	D4	A	3
S018	63	M	78	1.70	26.99	D9	D	3
S019	60	F	70	1.70	24.22	D12	B	41
S020	28	M	64	1.75	20.90	D7	B	2
S021	62	M	75	1.75	24.49	D8	A	20
S022	30	F	102	1.70	35.29	D4	D	13

M, male; F, female; BMI, Body Mass Index; AIS, ASIA Impairment Scale—indicates missing information.

**Table 2:** Delta cluster comparison of subjects with AB versus CD sense–motor capacities preserved after spinal cord injury and comparison of the two clusters with respect to the eyes open (OE) and eyes closed (CE) condition.

Posturographic Measure	Group	Visual Condition		*p*-Value
		OE	CE	
Total sway length (mm)	∆AB	75.46 (±30.60)	46.58 (±26.11)	0.0339 *
	∆CD	116.92 (±77.18)	71.52 (±31.82)	0.3750
	*p*-value	0.0905	0.0271 *	
Mean Velocity (mm/s)	∆AB	2.31 × 10^3^ (±1.5 × 10^3^)	2.35 × 10^3^ (±1.6 × 10^3^)	0.7148
	∆CD	4.10 × 10^3^ (±1.8 × 10^3^)	1.17 × 10^3^ (±688.22)	0.0101 *
	*p*-value	0.0327 *	0.0398 *	
SDC Mean Distance (mm)	∆AB	0.96 (±0.8)	0.82 (±0.7)	0.6620
	∆CD	2.02 (±1.3)	1.06 (±0.5)	0.2096
	*p*-value	0.03647 *	0.4548	
SDC Mean Time (s)	∆AB	0.08 (±0.05)	0.11 (±0.09)	0.3258
	∆CD	0.16 (±0.13)	0.1 (±0.06)	0.2166
	*p*-value	0.0796	0.9109	
SDC Mean Peak (s)	∆AB	0.43 (±0.31)	0.33 (±0.24)	0.2187
	∆CD	0.56 (±0.17)	0.52 (±0.29)	0.6630
	*p*-value	0.3094	0.1465	

∆ = delta; SDC = sway density curve; * Level of significance 95% when *p* < 0.05.

**Table 3:** Delta cluster comparison of subjects with a spinal cord injury affecting the fourth dorsal vertebra up to the cerebral vertebrae (C-D4) and those with an injury from the fifth dorsal vertebra downwards (D5-L) and comparison of the two clusters with respect to the eyes open (OE) and eyes closed (CE) condition.

Posturographic Measure	Group	Visual Condition		*p*-Value
		OE	CE	
Total sway length (mm)	∆(C-D4)	64.72 (±23.76)	40.90 (±16.78)	0.1030
	∆(D5-L)	106.48 (±58.54)	88.68 (±58.25)	0.3293
	*p*-value	0.0717	0.0370 *	
Mean Velocity (mm/s)	∆(C-D4)	2.68 × 10^3^ (±1.6 × 10^3^)	1.22 × 10^3^ (±503.41)	0.0156 *
	∆(D5-L)	3.22 × 10^3^ (±1.7 × 10^3^)	2.66 × 10^3^ (±1.7 × 10^3^)	0.3105
	*p*-value	0.2937	0.0367 *	
SDC Mean Distance (mm)	∆(C-D4)	0.81 (±0.58)	0.93 (±0.84)	0.7422
	∆(D5-L)	1.01 (±0.78)	0.95 (±0.54)	0.8043
	*p*-value	0.5262	0.4913	
SDC Mean Time (s)	∆(C-D4)	0.15 (±0.13)	0.13 (±0.07)	0.9999
	∆(D5-L)	0.08 (±0.04)	0.06 (±0.03)	0.2010
	*p*-value	0.2050	0.0105 *	
SDC Mean Peak (s)	∆(C-D4)	0.49 (±0.26)	0.40 (±0.23)	0.2403
	∆(D5-L)	0.42 (±0.25)	0.40 (±0.27)	0.8244
	*p*-value	0.5568	0.9748	

∆ = delta; SDC = sway density curve; * Level of significance 95% when *p* < 0.05.

## Data Availability

Dataset available upon request from the authors.
